# Associations between Reoperations and Psychological Factors after Contralateral Risk-Reducing Mastectomy: A Two-Year Follow-Up Study

**DOI:** 10.1155/2016/4604852

**Published:** 2016-05-26

**Authors:** Dmytro Unukovych, Marie Wickman, Kerstin Sandelin, Brita Arver, Hemming Johansson, Yvonne Brandberg

**Affiliations:** ^1^Department of Oncology-Pathology, Karolinska Institutet, 171 76 Stockholm, Sweden; ^2^Department of Reconstructive Plastic Surgery, Karolinska University Hospital, 171 76 Stockholm, Sweden; ^3^Department of Molecular Medicine and Surgery, Karolinska Institutet, 171 76 Stockholm, Sweden; ^4^Department of Breast and Endocrine Surgery, Karolinska University Hospital, Stockholm, Sweden; ^5^Department of Oncology, Karolinska University Hospital, 171 76 Stockholm, Sweden

## Abstract

*Introduction*. The aim of the study was to investigate associations between reoperations after contralateral risk-reducing mastectomies (CRRM) and emotional problems, body image, sexuality, and health related quality of life (HRQoL) in women with breast cancer and hereditary high risk.* Patients and Methods*. Patients scheduled for CRRM with breast reconstruction between 1998 and 2010 completed questionnaires, comprised of SF-36, the Hospital Anxiety and Depression Scale, the Body Image Scale, and the Sexual Activity Questionnaire, preoperatively and two years after CRRM. Data on reoperations was collected from medical charts.* Results*. A total of 80 women participated, with a response rate of 61 (76%) preoperatively and 57 (71%) at the two-year follow-up. At the two-year assessment, 44 (55%) patients had undergone ≥1 reoperation (reoperation group), whereas 36 (45%) had not (no reoperation group). No statistically significant differences between the groups were found for HRQoL, sexuality, anxiety, or depression. A higher proportion of patients in the “reoperation group” reported being dissatisfied with their bodies (81% versus 48%, *p* = 0.01).* Conclusion*. The results suggest associations between reoperation following CRRM with breast reconstruction and body image problems. Special attention should be paid to body image problems among women who are subject to reoperations after CRRM.

## 1. Introduction 

Risk-reducing mastectomy (RRM) is an established preventive measure for women at high risk of breast cancer. In general, studies have shown that women are satisfied with the outcomes of RRM including reconstruction, and similar levels of health related quality of life (HRQoL) as before the operation have been reported [1–3]. Problems with body image and sexuality have, however, been identified in several studies of RRM. In one study, 487 completed a questionnaire at a mean of 20 years after CPM. The women reported that body appearance (31%), feelings of femininity (24%), and sexual relationships (23%) were most affected [4]. A majority reported pain and discomfort in the breasts, as well as reduced sexual sensations, in a two-year follow-up of 59 women undergoing bilateral risk-reducing mastectomy due to increased hereditary risk of breast cancer [5]. A long-term follow-up (6–9 years) after prophylactic mastectomy and breast reconstruction in 36 women at high risk of breast cancer revealed persistent problems regarding body image [6]. Hopwood and coworkers [7] found no evident problems with body image among the majority of 76 women three years after RRM but concluded that those who had complications might warrant psychological support. A review of thirteen studies of body image after RRM concluded that the majority of studies indicated that almost half of the women reported a negative effect on body image and changes in sexuality [8].

Patients who have been diagnosed with breast cancer and identified with hereditary high risk are informed of the possibility of considering a contralateral risk-reducing mastectomy (CRRM) with or without breast reconstruction. CRRM is, in most cases, performed when the primary cancer treatment is accomplished and an ipsilateral preventive and/or reconstructive intervention is considered. We have previously shown that the reconstructive procedure is challenging and may require adjustments and reoperations, especially on the cancer side that has often been irradiated [9]. Thus, more than half of the patients receiving CRRM with bilateral breast reconstruction in that study were reoperated during four years of follow-up. A second study showed no difference in HRQoL pre- versus postoperatively, but specific concerns and dissatisfaction with body image were reported postoperatively [10].

Due to the fact that reoperations, as well as problems with sexuality and body image, following CRRM are common we assumed that the reported problems could be associated with the surgical procedures, hypothesising that those with reoperations would report lower levels of HRQoL and higher levels of body image associated problems.

The aim of the present study was to investigate differences in HRQoL and problems with body image and sexuality between women with high hereditary risk of breast cancer who underwent reoperations and those who did not during two years after CRRM.

The study was approved by the Ethics Committee at Karolinska Institutet (Dnr 2005/685-31).

## 2. Patients and Methods

### 2.1. Patients

During 1998–2010, patients scheduled for CRRM at the Karolinska University Hospital were invited to participate in a prospective study of HRQoL, emotional reactions, sexuality, and body image. Patient recruitment and data collection have been described in detail elsewhere [9, 10]. The patients were asked to complete a study questionnaire preoperatively, as well as six months and one and two years after CRRM. One reminder was sent by post at each assessment point if no response was received after two weeks. In the present paper, the assessment points preoperatively and two years after CRRM were used, as the aim was to investigate if undergoing a reoperation during the total CRRM procedure was associated with HRQoL, sexuality, and body image. The last point of questionnaire collection was two years after CRRM procedures. The previous study of reoperations had a follow-up time of four years and was based on data from 91 patients operated during 1998–2008 [9]. The second study focusing on HRQoL after 2-year follow-up included 61 of the patients from the first study [10]. In the present paper 19 patients were added, also with a two-year follow-up time after CRRM in order to match the time point of the last questionnaire collection.

### 2.2. Contralateral Risk-Reducing Mastectomy with Immediate Breast Reconstruction

Skin-sparing mastectomy with immediate implant-based breast reconstruction was performed in all cases. The surgical technique used at the Karolinska University Hospital for the procedure has been described in detail previously [9, 10]. We have almost exclusively used implant reconstructions also in the reoperations.

### 2.3. Clinical Data Collection

Clinical data (age, BRCA mutation status, prophylactic salpingo-oophorectomy or not, primary tumor size, lymph nodes, radiotherapy or not, chemotherapy or not, endocrine therapy or not, CPM side, reconstruction method, and simultaneous operation on cancer side) and dates for reoperations were collected from patients' files.

Reoperation was defined as an unanticipated intervention on any breast following CRRM with breast reconstruction requiring operation under general anaesthesia, that is, implant removal/replacement, capsulotomy/capsulectomy, early revisions due to, for example, bleeding or infection, or others.

### 2.4. Questionnaires

The study questionnaire comprised four validated patient reported outcomes measures.

*The Body Image Scale* (BIS) assesses, by ten items, the impact of surgery on patients' self-consciousness, physical and sexual attractiveness, femininity, satisfaction with body and scars, body integrity, and avoidance behaviour. The sum of the BIS items gives an overall score [11].

*The Sexuality Activity Questionnaire* (SAQ) includes ten items assessing sexual activity and is divided into three sections: pleasure (desire, enjoyment, and satisfaction), discomfort (dryness, pain), and habit (sexual behaviour) [12]. The pleasure section includes 6 items (scores: from 0 to 18), the discomfort section two items (scores: from 0 to 6), and the habit section one item (scores: from 0 to 3). The sum of all items within each section produces the three overall scores.

*The Hospital Anxiety and Depression Scale* (HADS) was designed to detect clinically relevant anxiety and depression in somatically ill patients, excluding items of somatic symptoms of anxiety and depression. It consists of 14 items constituting two subscales, the anxiety subscale (7 items) and the depression subscale (7 items). Cut-off levels for clinical levels of anxiety and depression have been specified [13]. The Swedish version of the HADS has been validated against diary ratings in breast cancer patients [14].

*The Medical Outcomes Study 36-Item Short Form* (SF-36) is a self-administered questionnaire evaluating general health status and generic health concepts not specific to age, disease, or treatment group [15]. SF-36 includes 36 items, defining eight HRQoL domains: physical functioning, role limitations due to physical problems, bodily pain, social functioning, general mental health, role limitations due to emotional problems, vitality, and general health perception. The questionnaire has been translated into many languages with further validation in multicultural settings [16].

## 3. Statistical Analyses

STATA/SE (Version 13; StataCorp, TX, USA) was used for all statistical analyses. Tests for differences between patients with and without reoperations in clinical and demographic characteristics were performed using Fisher's exact test.

All single items in the SF-36 were transformed into the eight subscales with scores ranging from 0 to 100 [16]. High figures represent higher level of functioning and HRQoL. Clinical significant differences were determined according to Osoba and coworkers [17] as follows: <5 = not clinically significant, 5 to 9 = small clinical significance, 10 to 19 = moderate clinical difference, and ≥20 = large clinical significance.

For the BIS subscales presentation, we dichotomized the results into 0 (no problems) and 1–3 (problems or negative changes). Mean scores for SF-36, HADS, BIS, and SAQ at the postoperative assessment were analysed using linear regression models and adjusted for baseline (i.e., preoperative) levels and radiotherapy.

The level of statistical significance was set to 0.01 due to multiplicity.

## 4. Results

A total of 125 women were scheduled for CRRM during the study period, and 80 patients (64%) were invited to participate in the questionnaire study. The reason for not being invited was administrative failure. The response rates preoperatively and two years after the operation were 61 (76%) and 57 (71%), respectively.

Mean age at CRRM in the cohort was 44 years (range: 25–65). No contralateral breast cancer was registered during the two-year follow-up period. Seven patients were diagnosed with distant metastases from their primary breast cancer after the date of CRRM, of whom six died of breast cancer.

During the two-year study follow-up from CRRM 44 (55%) patients underwent one or more reoperations (reoperation group) and 36 (45%) did not require any reoperation (no reoperation group). Clinical and demographic characteristics of the participants are presented according to group (Table 1).

**Figure 1:**
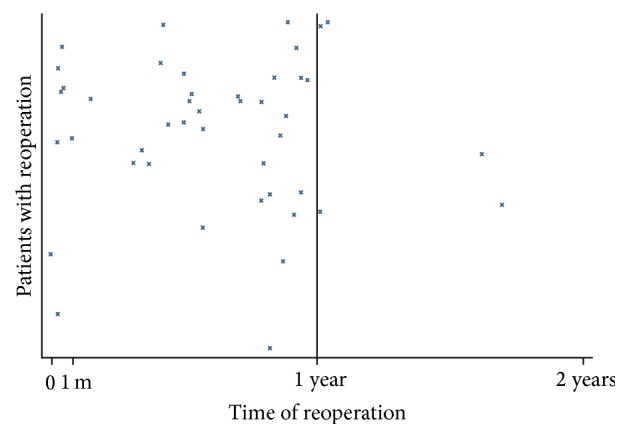
Timing of reoperations following contralateral risk-reducing mastectomy.

Between-group comparisons revealed that the “reoperation group” received radiotherapy more often than the “no reoperation group,” 37 patients (84%) versus 21 (57%), respectively (*p* = 0.005). In the reoperation group, 7/44 (16%) women required reoperation within one month postoperatively, and the remaining 37/44 (84%) underwent adjustments at a later time point. The time points for reoperation related to the time of CRRM are presented for each patient in Figure 1.

### 4.1. Body Image Scale (BIS)

**Figure 2:**
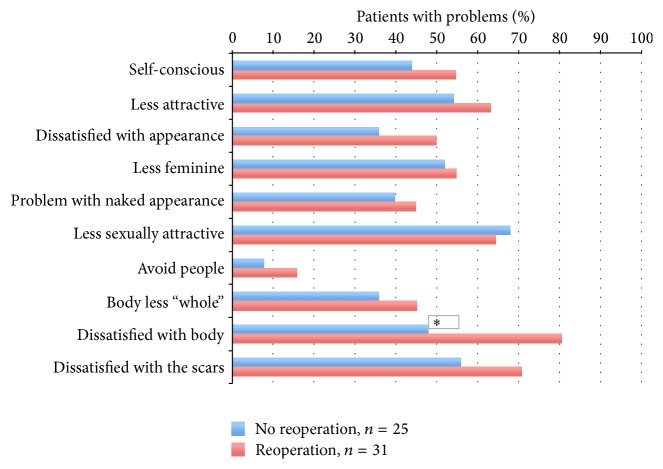
Percentage of patients reporting problems with body image two years after CRRM. ^*∗*^ *p* = 0.010 (adjusted for radiotherapy).

When comparing the proportions of patients having problems with the BIS items, a statistically significant difference in “dissatisfaction with the body” (81% versus 48%, *p* = 0.01) appeared, revealing more problems in the “reoperation group” than in the “no reoperation group” (Figure 2). Higher proportions of patients in the “reoperation group” than in the “no reoperation group” reported problems with eight out of nine items on the BIS scale, although not statistically significant. No notable changes in the results were found when adjusted for radiotherapy (data not shown).

### 4.2. Sexual Activity Questionnaire (SAQ)

No between-group differences were found with respect to sexual pleasure, discomfort, or habit (Table 2).

### 4.3. Anxiety and Depression Scale (HADS)

There were no statistically significant differences found in anxiety or depression between the groups two years after CRRM (Table 2).

### 4.4. Health Related Quality of Life (SF-36)

Two years after CRRM, no statistically significant differences were found between the groups with respect to any of the SF-36 variables. After adjustment for radiotherapy, moderate clinical differences in “bodily pain” and “role emotional” subscales were found, with higher scores in the “no reoperation group” (Table 3). Small clinical differences were, however, found for “role physical” and “general health,” favouring the “reoperation group.”

## 5. Discussion

As problems with body image and sexuality are frequently reported following RRM with breast reconstruction, we wanted to evaluate if additional surgical procedures in women with a breast cancer diagnosis could be associated with these problems. Studies of psychosocial implications of reoperations in patients undergoing prophylactic mastectomy are sparse in the literature.

In the present study, women who had reoperations appeared to have more problems with body image than women without reoperation. One explanation for these findings might be that reoperations affected the cosmetic outcome negatively. In addition, the fact that reoperations were needed indicates that the cosmetic results after CRRM might have been less satisfactory. Notably, negative appraisal in body image was apparent in both groups (Figure 2), which may be related to issues other than those associated with the reoperation, for example, the mastectomy itself, breast implants, postoperative scars, loss of breast sensation, and appearance of the nipple-areolar complex.

Patients in the “reoperation group” received radiotherapy more often than the “no reoperation group” (*p* = 0.005). Interestingly radiotherapy, when included in the multivariate model, did not appear to have impact on body image. Long-lasting side effects after radiotherapy are, however, relatively common and might make breast reconstruction more complicated, probably resulting in the need for reoperations. When the results remain unfavourable more complex reconstructive techniques should be discussed with the patient when planning for further operations.

“Bodily pain” and “role emotional” were “moderately” clinically better in the “no reoperation group.” A possible explanation for less “bodily pain” in the “no reoperation group” is that they have been subjected to less surgery. The worry evoked by having to undergo more surgery might explain the lower levels of “role emotional” in this group. On the other hand, “role physical” and “general health” showed a small clinical difference, favouring the “reoperation group.” The reason for the clinical difference might be the tendency of a lower proportion of women in the “reoperation group” who received chemotherapy, probably still affecting the physical role functioning and feeling of general health. In a Swedish study, women two to five years after a breast cancer diagnosis reported lower levels of role functioning and global quality of life than healthy controls of the same age [18].

No statistically significant differences were found for sexuality, anxiety, and depression, despite the finding of more problems with body image in the “reoperation group.” Both groups appeared to have sex less frequently than usual according to the responses to the question about habit in SAQ. It is likely that factors other than problems with body image contribute to the frequency of sex, for instance, hormonal treatment.

We found only two papers focusing on HRQoL and psychosocial aspects in patients experiencing complications after breast reconstruction. One study assessed 60 patients who underwent latissimus dorsi reconstructions with (*n* = 25) and without (*n* = 35) early surgical complications [19]. No differences were found for EORTC QLQ-C30 or FACT at the three-month postoperative assessment. Women with complications, however, were more often dissatisfied with the scars and reported feeling less feminine than those without complication. The high rate of complications in that study might be explained by the fact that the latissimus dorsi procedure leaves larger scars and sometimes donor site morbidity. The follow-up time in that study was only three months, and in our study sample, reoperations due to early surgical complications were rare. Most of the adjustments were performed to improve the aesthetic outcome of the breast reconstruction.

In another study, patients undergoing breast reconstruction with implants (*n* = 71) or DIEP flaps (*n* = 81) were asked about surgical complications as well as cancer-specific distress, anxiety, and depression [20]. Four weeks postoperatively 35% of the patients in the implant group and 46% in the DIEP group reported complications (i.e., self-reported complications). Patients who perceived complications reported more anxiety and depressive symptoms, whereas cancer-specific distress appeared not to be related to complications and generally decreased postoperatively. This study had an even shorter follow-up, and it is likely that the problems experienced were related to the ongoing reoperation complications. It might, however, be assumed that women who report anxiety and depressive symptoms perceive self-reported complications to a higher extent than those who do not experience these problems.

The strengths of the present study lie in its prospective design, where consecutive patients participated, and in that standardized instruments were used preoperatively and in the long-term follow-up. In addition, all medical charts were reviewed for complications and reoperations until the end of study follow-up. Thus, the collected data are considered solid. One weakness of the study is the administrative failure, resulting in that only 64% of the eligible patients were included. In addition, the response rate was relatively low (between 76 and 71%) at both points of assessment. It should be remembered that the study was conducted in a clinical context.

In conclusion, the results suggest that reoperations following CRRM with breast reconstruction are associated with long-term body image problems. No associations were, however, found between reoperations and emotional reactions, sexuality, or HRQoL. Special attention should be paid to body image problems among women who are subject to reoperations after RRM.

## Figures and Tables

**Table 1:** Demographic and clinical data for all eligible patients according to reoperation after CRRM (*n* = 80).

Variable	No reoperation *n* = 36 (%)	Reoperation^#^ *n* = 44 (%)	*p* value^*∗*^
Age at CPM, years			
25–35	5 (13.9)	7 (15.9)	0.85
35–45	13 (36.1)	19 (43.2)	
45–55	11 (30.6)	12 (27.3)	
55–65	7 (19.4)	6 (13.7)	
*BRCA* mutation status			
* BRCA1*	16 (44.4)	18 (40.9)	0.14
*BRCA2*	7 (19.5)	2 (4.6)	
*BRCAX* ^a^	9 (25.0)	17 (38.6)	
No mutation or unknown	4 (11.1)	7 (15.9)	
Prophylactic salpingo-oophorectomy			
Yes^b^	20 (55.6)	16 (36.4)	0.09
No	16 (44.4)	28 (63.6)	
Primary tumor size			
Tis	4 (11.1)	2 (4.6)	0.61
T1	18 (50.0)	25 (56.8)	
T2	10 (27.8)	14 (31.8)	
T3	4 (11.1)	3 (6.8)	
Lymph nodes			
N+	18 (50.0)	23 (52.3)	0.84
N0	18 (50.0)	21 (47.8)	
Radiotherapy			
Yes	20 (56.6)	37 (84.1)	0.005
No	16 (44.4)	7 (15.9)	
Chemotherapy			
Yes	19 (52.8)	33 (75.0)	0.038
No	17 (47.2)	11 (25.0)	
Endocrine therapy			
Yes	19 (52.8)	23 (52.3)	0.96
No	17 (47.2)	21 (47.7)	
Side CPM			
Right	23 (63.9)	21 (47.7)	0.30
Left	13 (36.1)	21 (52.3)	
Reconstruction with			
Permanent implant	15 (41.7)	12 (27.3)	0.18
Expandable implant	21 (58.3)	32 (72.7)	
Simultaneous operation on cancer side			
Delayed reconstruction	16 (44.4)	16 (36.4)	0.59
Completion mastectomy^$^ with IBR^c^	12 (33.4)	20 (45.5)	
Implant replacement	4 (11.1)	4 (9.1)	
No operation	4 (11.1)	4 (9.1)	

CRRM: contralateral prophylactic mastectomy; IBR: immediate breast reconstruction.

^#^Unanticipated surgical procedure on any breast after CRRM with breast reconstruction.

^*∗*^Fisher's exact test.

^$^Removal of the remaining breast after previous breast-conservative surgery.

^a^Patient screened negative for known mutations having any other case of BC onset before the age of 50 in the family.

^b^Five participants underwent prophylactic salpingo-oophorectomy after CPM.

^c^One patient underwent therapeutic mastectomy.

**Table 2:** Postoperative scores and standard deviation (SD) for BIS, SAQ, and HADS and differences between the “reoperation group” and the “no reoperation group.”

Questionnaire and subscales	Mean (SD)		Crude diff. (99% CI)	*p* value	Adjusted diff.^*∗*^ (99% CI)	*p* value^*∗*^
	No reoperation *n* = 16–24	Reoperation *n* = 25–30				
BIS						
Summated score^a^	6.3 (5.6)	7.8 (5.5)	−1.5 (−5.5 to 2.6)	0.34	−1.5 (−6.0 to 3.1)	0.39
SAQ						
Pleasure^b^	9.3 (5.0)	9.5 (4.3)	−0.2 (−4.2 to 3.8)	0.91	−0.4 (−4.8 to 3.9)	0.78
Discomfort^c^	1.2 (1.4)	1.8 (1.9)	−0.6 (−2.2 to 0.9)	0.25	0.8 (−0.6 to 2.3)	0.12
Habit^d^	0.7 (0.5)	0.9 (0.9)	−0.2 (−0.8 to 0.4)	0.36	0.3 (−0.6 to 1.1)	0.39
HADS						
Anxiety^e^	5.5 (4.4)	5.7 (3.8)	−0.2 (−3.2 to 2.8)	0.86	−0.7 (−4.2 to 2.8)	0.58
Depression^e^	2.8 (2.8)	3.0 (3.2)	−0.2 (−2.4 to 2.0)	0.81	−0.5 (3.1 to 2.2)	0.64

BIS: Body Image Scale; SAQ: Sexual Activity Questionnaire; HADS: Hospital Anxiety and Depression Scale; CI: confidence interval.

^a^Higher score indicates more problems (range 0–30).

^b^Higher scores indicate more pleasure (range 0–18).

^c^Higher scores indicate more discomfort (range 0–6).

^d^Score < 1 indicates less frequency than usual (range 0–3).

^e^Higher scores indicate higher levels of anxiety and depression (range 0–21).

^*∗*^Adjusted for radiotherapy.

**Table 3:** Health related quality of life two years after contralateral prophylactic mastectomy.

SF-36 subscale	Mean (SD)		Crude diff. (99% CI)	*p* value	Adjusted diff.^*∗*^ (99% CI)	*p* value
	No reoperation *n* = 25	Reoperation *n* = 31				
Physical functioning	90 (16)	86 (20)	4 (−9 to 18)	0.39	4 (−11 to 19)	0.47
Role physical	78 (39)	82 (29)	−4 (−29 to 20)	0.64	−8 (−36 to 20)^a^	0.44
Bodily pain	84 (24)	77 (28)	7 (−12 to 26)^a^	0.33	13 (−9 to 35)^b^	0.12
General health	71 (25)	77 (20)	−6 (−22 to 10)^a^	0.29	−7 (−19 to 6)^a^	0.15
Vitality	65 (25)	64 (23)	1 (−17 to 18)	0.94	−3 (−23 to 16)	0.65
Social functioning	89 (21)	84 (21)	5 (−11 to 20)^a^	0.42	4 (−16 to 25)	0.57
Role emotional	87 (32)	74 (40)	13 (−14 to 39)^b^	0.21	11 (−17 to 39)^b^	0.29
Mental health	77 (17)	73 (24)	4 (−11 to 19)	0.50	−1 (−20 to 19)	0.92

^a^Small clinically significant difference (5 to 9 points).

^b^Moderate clinically significant difference (10 to 19 points).

^*∗*^Adjusted for radiotherapy.
